# Improved scheduling algorithm for signal processing in asynchronous distributed ultrasonic total-focusing-method system

**DOI:** 10.1371/journal.pone.0212285

**Published:** 2019-02-14

**Authors:** Yuzhong Li, Wenming Tang, Guixiong Liu

**Affiliations:** 1 School of Mechanical & Automotive Engineering, South China University of Technology, Guangzhou, China; 2 School of Information Engineering, Huizhou Economic and Polytechnic College, Huizhou, China; 3 Guangzhou Doppler Electronic Technologies Co., Ltd., Guangzhou, China; Chang Gung University, TAIWAN

## Abstract

Compared to the conventional ultrasonic phased-array system, a large-element phased-array system employing the total focusing method (TFM) can yield improved image resolution and accuracy, providing more flexible scanning methods and image merging functionality. In order to meet various forms of ultrasonic multi-group scanning, an architecture for multi-group scan integration called the “asynchronous distributed ultrasonic TFM system” is proposed, and a novel scheduling algorithm called “the sum of start time and processing time adjacent (SSPA) algorithm” is presented. The architecture adds a focus and group scheduler (FGS) and signal processing scheduler (SPS) to the traditional ultrasonic phased array system and constructs a signal processing arbitration (SPA) with several signal processing modules (SPMs). The FGS provides the focus parameters, pixel memory range, and number of pixels in each group. The SPS controls the SPA for the ultrasonic scanning data obtained from the elements, with SPM-sharing output data; hence, the optimal priority order and SPM assignment are realized, enabling switching of reading operations among the first-in−first-out memories for signal processing and minimal time-slot waiting. The SSPA algorithm is used to solve the job-shop scheduling problem with start time, which considers the processing time and start time, in order to reduce the time slot after each scheduling using adjacent operations. Therefore, the architecture enhances the flexibility of the multi-group scan, and this algorithm decreases the makespan, achieving higher efficiency compared to conventional scheduling algorithms. The reliability and validity of the algorithm are substantiated after its implementation using FPGA technology. The SPM utilization rate and the real-time performance of the ultrasonic TFM are improved. Thus, the proposed algorithm and architecture have considerable potential application in multi-sensor systems.

## Introduction

Recently, techniques associated with multi group ultrasonic sensors, which include numerous piezoelectric elements and various ultrasonic phased array (UPA) scanning patterns, have attracted widespread attention in the field of nondestructive testing [1,2].

**Fig 1:**
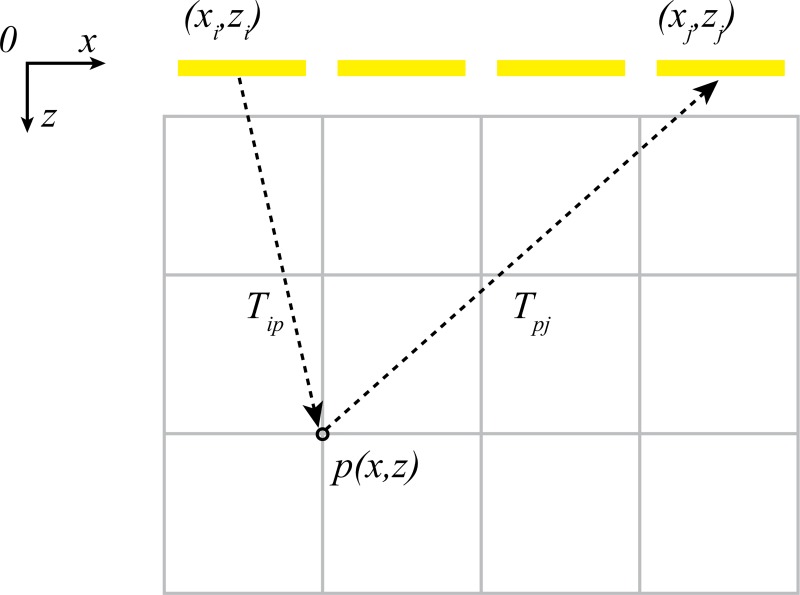
Total focusing method concept.

The full matrix capture-total focusing method (FMC-TFM) is a high-resolution imaging technique used in UPA systems, which was proposed by Holmes et al. in 2005 [3]. Full matrix capture (FMC) acquisition obtains the most complete imaging information for subsequent processing by acquiring all the transmit-receive pairs of the A-sweep dataset in multiple ultrasonic sensors. Fig 1 depicts the concept behind the total focusing method (TFM) algorithm. This algorithm meshes the region of interest (ROI) in a grid of pixels; a pixel is generated by the summation of the data from all the transmission-reception pairs. [3] The intensity, *I*, of a pixel, *P(x*, *z)*, is expressed as

$$I[P\left(x,z\right)]=\left|\sum_{i=1}^{N_{e}}\sum_{j=1}^{N_{e}}h_{ij}\left(T_{ip}+T_{pj}\right)\right|,$$ (1)

where *h*_*ij*_ is the analytical version of the echo received by element *j* when element *i* transmits; $T_{ip}+T_{pj}=\left(\sqrt{{\left(x_{i}-x\right)}^{2}+z^{2}}+\sqrt{{\left(x_{j}-x\right)}^{2}+z^{2}}\right)/c$ is the time of flight between *P* and the element pair (*i*, *j*); *N*_*e*_ is the number of elements; *x*_*i*_ and *x*_*j*_ are the coordinates of elements *i* and *j*, respectively; and *c* is the longitudinal velocity of sound.

To increase the focusing ability, the UPA instrument is often equipped with multiple ultrasonic sensors to collect the ultrasonic echo data from different directions. Sensors can work in one or more groups to generate a variety of scanning modes [4–8]; this is called multi group scanning, and each group scan includes several focused beams. The advantage of multi group scanning is improved scan flexibility through grouping, and realization of image merging[9–11]. For example, Song et al. verified that a large-aperture hemispherical phased array can restore sharp focus and maximize acoustic-energy delivery to target tissue [12]. However, this strategy increases the scanning data considerably in the defect detection process [13], rendering it difficult to transmit these data to a signal-processing module (SPM) for subsequent production of an ultrasound image.

Therefore, an asynchronous distributed ultrasonic system that applies the TFM method is proposed in this work. The proposed “asynchronous distributed ultrasonic TFM system” meets the following requirements: (i) distributed scanning can be realized, with each scanning group having potentially different start times, sample depths, and pixel synthesis parameters; (ii) compared to conventional arrays, more echo data can be derived by using an array with a larger number of sensor elements, thus achieving better detection resolution; (iii) asynchronous sampling and scanning can alleviate the requirement for a synchronous clock in the field-programmable gate array (FPGA); (iv) integrated focus module and SPMs in a single FPGA for the TFM can process the echo data in the same domain, reducing the system complexity and solving the clock jitter between scan groups; and (v) the FMC-TFM system can flexibly group the array without restricting the number of elements in each group.

However, asynchronous distributed ultrasonic TFM systems have certain problems that must be addressed: (i) multi group scanning involves different focal laws, scanning depths, and sound velocities, and the distributed architecture also has different start times, data numbers, and focus parameters; this yields different frame tasks (beam forming image data) with different numbers of data elements, and the arrival of these tasks may result in time overlap; (ii) limited by the FPGA in a single chip resource, the system cannot build SPMs for each element; however, it should realize real-time image display, which necessitates an SPM scheduling mechanism; (iii) in a distributed image combination application, the FPGA must ensure that several images are focused and processed in real time, within a 0.04-s period, to ensure real-time image combination.

As a result of the above-mentioned reasons, the asynchronous distributed TFM ultrasonic system needs to schedule the pixel data in first-in−first-out memories (FIFOs) before the data enter the SPM, to ensure correct and orderly pixel-data processing. To overcome a large number of problems associated with asynchronous focusing data and its scheduling, the “sum of start time and processing time adjacent (SSPA) algorithm” is also proposed in this paper. This algorithm performs multiplexing of the SPMs, maximizes the resource utilization, and ensures real-time performance.

The remainder of this paper is organized as follows: In the section Related Work, we discuss the related work of the job-shop scheduling problem (JSSP), FPGA parallel architecture and application, the distributed ultrasonic system, and multi-group scanning in an ultrasonic system. In the section Problem Description, we describe the target problem. The architecture of the asynchronous distributed ultrasonic TFM system, and the signal processing scheduling problem for multi-group scanning and its mathematical model are discussed. In the section Proposed SSPA Algorithm, we study the scheduling mechanism of the SSPA algorithm. In the section Experiment Results and Discussion, we present the results of an experiment comparing the first come, first served (FCFS) and shortest processing time (SPT) algorithms with the proposed SSPA scheduling algorithm implemented using the FPGA technology. Finally, in the section Conclusions and Future Research, we summarize the research and derive conclusions.

## Related work

The SPM scheduling algorithm in the asynchronous distributed TFM ultrasonic system involves a job-shop scheduling problem with a start time (JSSP with ST). As the scheduling algorithm is intended to ensure real-time imaging, it cannot involve considerable calculation, and the algorithm flow and structure need not only ease the migration to the FPGA, but also avoid the impact of pseudo random numbers by random algorithm.

The research methods of the JSSP can be divided into two categories: optimization method and approximate/heuristic method. The optimization method mainly includes mixed integer linear programming, branch and bound and Laplace relaxation. Approximate/heuristic algorithms were originally introduced into JSSP problems because of their low computational complexity and easy implementation[14,15]. It mainly includes priority dispatching rules (PDRs), artificial intelligence, neural network, and neighborhood search method. The neighborhood search method includes tabu search, genetic algorithm, and simulated annealing, which is the meta-heuristic approximate optimization method. The earliest PDR was presented by Johnson [16] and Smith [17]. Other PDRs for the JSSP include the SPT, longest processing time, most work remaining, least work remaining, most operation remaining, least operation remaining, earliest due date (EDD), and selection of the first procedure in the work piece queue on the same machine (i.e., FCFS)[18]. Panwalkar [19] presented a summary of approximately 113 dispatch rules, classified through performance indexes. Furthermore, Wu [20] stated that scheduling rules can be divided into three categories: priority rules related to the job information, a combination of priority rules and switching, and the weighted priority dispatching rules. The key lies in selecting the best rule for a given problem. As per the optimization effect of each rule, the SPT can reduce the average process time of all the jobs, and the EDD is used for optimizing the target related to the maximum delay. Previously, Ying et al. [21] investigated no-wait flowshop scheduling problems with sequence-independent (NWFSP with SISTs) and sequence-dependent setup times (SDSTs) with the aim of minimizing the makespan. Hence, they proposed an efficient two-phase matheuristic. Their study is latest and the proposed TPM algorithm has achieved high performance. Furthermore, Lin et al. [22] investigated the system testing scheduling problem and used a method applicable to a computer manufacturing company; however, they used the algorithm in computer production. The JSSP with the ST generated by the ultrasonic multi-group scan system is not large in terms of the number of tasks, and the PDR of the approximate/heuristic method can reduce the calculation of the dispatch parameters and increase the scan verification time. Therefore, the SSPA algorithm adopts an improved PDR as a part of the algorithm.

In recent years, a large number of FPGA parallel architectures have been reported. Suzuki et al. [23] have proposed an FPGA architecture and implementation for a shared synapse architecture for autoencoders; this architecture utilizes less of the limited resources of an FPGA than an architecture that does not share the synapse weights, and it reduces the amount of synapse modules used by half. Rodríguez-Flores et al. [24] have proposed the evaluation of a scalable low-area FPGA hardware architecture for security protocols relying on public key encryption; the design can process operands of different sizes using the same data path, which exhibits a significant reduction in the area without loss of efficiency. Hossain et al. [25] have developed a novel parallel architecture for fast hardware implementation of elliptic curve point multiplication. The area-time product of the proposed point multiplication is low, and the performance product of the proposed design is improved. Kim et al [26] have suggested a pipelined non-deterministic finite automaton-based string matching scheme using FPGA implementation. By cutting down the number of used LUTs for implementing state transitions, the hardware overhead of combinational logic circuits is reduced. All the above parallel architectures are RTL-level to reduce area and resource usage, and asynchronous distributed signal processing module scheduling is not discussed.

Multiple examples of scheduling algorithm implementation are available in the literature [27–37]. Among the relevant achievements, Srinivasan and Pandharipande [27] designed a self-configuring scheduling protocol for ultrasonic sensor systems using a timeslot allocation algorithm, which simplified the deployment of ultrasonic sensor systems. Further, Long et al. [32] proposed a time-division-multiple-access-based energy consumption balancing algorithm for general k-hop wireless sensor networks, where one data packet is collected in a cycle; the results demonstrated the effectiveness of the algorithm in terms of the energy efficiency and timeslot scheduling. A distributed ultrasonic sensor system has also been used in many application scenarios [36–40]. For example, Caicedo and Pandharipande [36,38,40] have presented ultrasonic array sensor solutions for reliable presence detection in indoor spaces. In addition, Priyantha et al. [37] have presented the design, implementation, and evaluation of the cricket location-support system for in-building, mobile, and location-dependent applications. Furthermore, Zhang et al. [39] have proposed a dynamic distributed sensor scheduling scheme, where the tasking sensor is elected spontaneously from sensors with pending sensing tasks via random competition based on carrier sense multiple access. Although the above implementations are effective in different distributed environment, application in the UPA system has not been discussed, and the distributed algorithm of its internal signal processing has not been studied. Therefore, in this work, we focus on the distributed UPA system and its algorithm.

## Problem description

### Asynchronous distributed ultrasonic TFM system architecture

**Fig 2:**
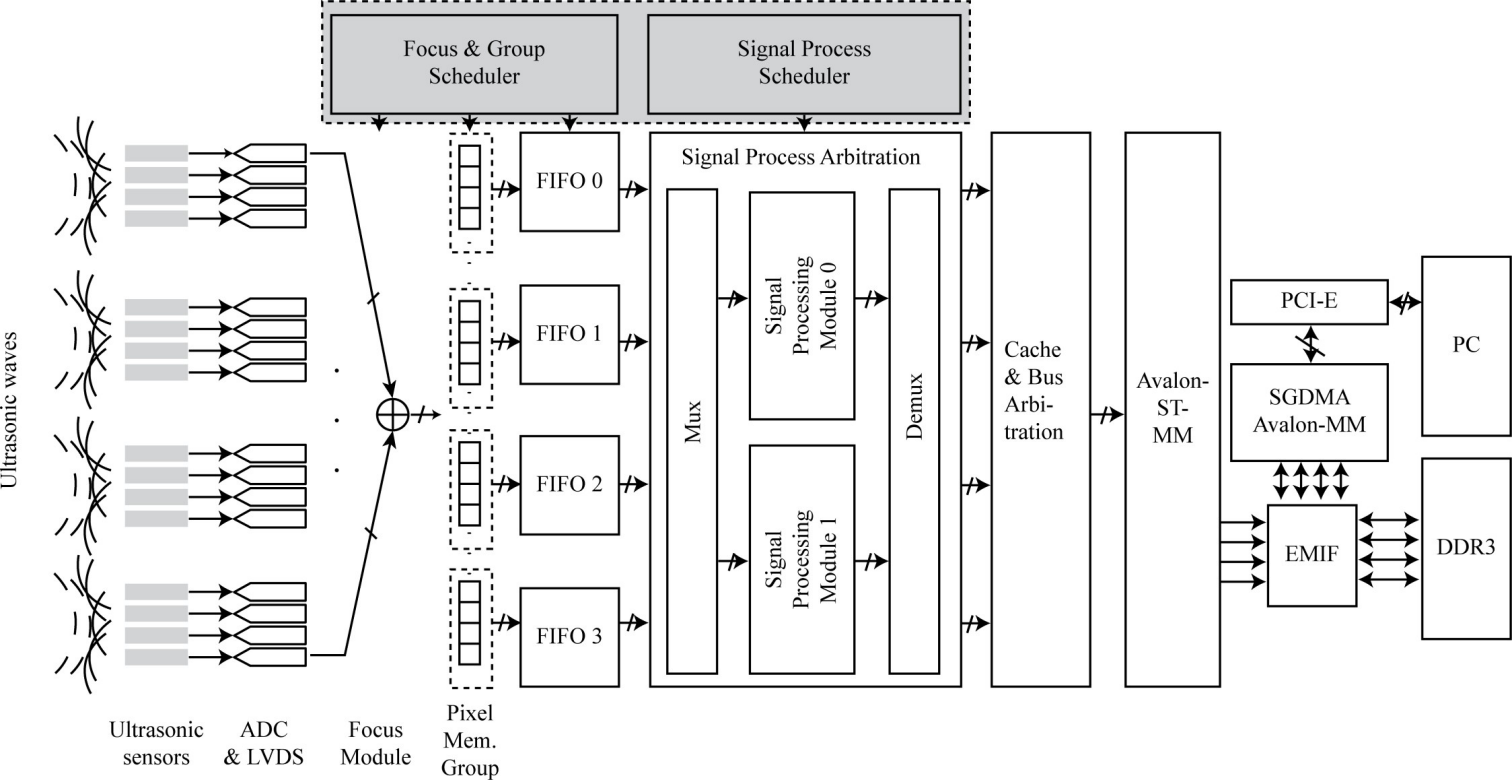
Architecture of asynchronous distributed ultrasound TFM system.

Fig 2 displays a block diagram of the asynchronous distributed ultrasound TFM system, which has four sensor groups and two SPMs. To realize integration of multi group scan systems, the signal processing scheduler focus and group scheduler (FGS) and the signal processing scheduler (SPS) are crucial components of this architecture. The FGS focuses the echo data from the analog-digital converter (ADC) and low-voltage differential signaling (LVDS) and saves the focused data in the corresponding location in the pixel memory group. The SPS schedules the signal data process in each signal process module. The other components include the ADC and LVDS, pixel memory group, focus module, FIFOs, signal processing arbitration (SPA), cache and bus arbitration, Avalon streaming to memory map (Avalon ST-MM), external memory interface (EMIF), scatter-gather direct memory access (SGDMA), peripheral component interconnect express (PCI-E), double-data-rate three synchronous dynamic random access memory (DDR3 SDRAM), and a personal computer (PC).

The signal flow is as follows: the ultrasonic emission signal is sent to the test block, the ultrasonic probe group receives the echo signal, and after ADC, it enters the focus module. The focus module is controlled by the FGS. When the scan group begins to scan, the focus module receives the signal from the ADC of the corresponding group and sends it to the assigned position of the pixel memory group after focusing. The FGS provides two types of information: the focus time parameter and the grouping parameters. The focus time parameter gives the delay time of each pixel, which is calculated from the TFM equation. The grouping parameters determine the scan group elements to be scanned, the receiving elements of the group, and the pixel position in the pixel memory group. After focusing, the readouts of multiple FIFOs are controlled by the signal process scheduler (SPS), which contains scheduling information on the signal processing order and SPM allocation. If the FIFOs have loaded the pixel data that have been focused by the focus module and an interrupt signal is transmitted, the data transmission waits for the signal-processing scheduler to prioritize the incoming SPM. After the SPM is processed, the data transmission waits for bus arbitration and dispatch. Finally, the data are sent to the PC through the PCI-E bus (S1 Appendix).

**Fig 3:**
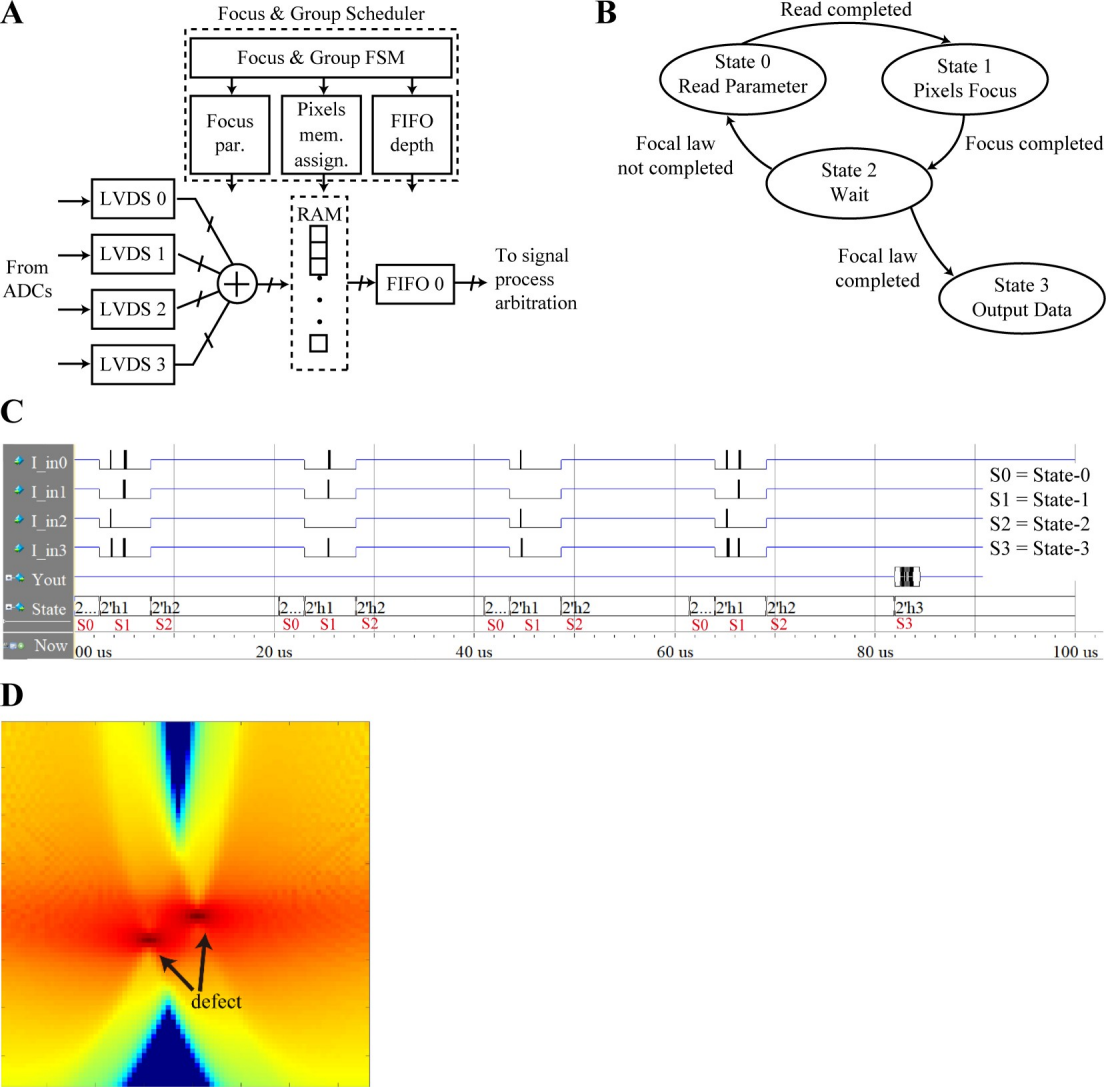
TFM scan and focus for group. (a) Flowchart, (b) architecture, (c) ModelSim simulation, and (d) focused image.

Next, we describe the FGS and SPS in detail, by considering one of the sensor groups in the system and their corresponding modules. Two scheduling parameters are introduced: the start time and processing time. The FGS and its corresponding module with four elements in a sensor group, shown in Fig 3(A), includes the focus parameter, pixel memory assign, and FIFO depth modules. The focus parameter module provides the focus parameter, the pixel memory assign provides the pixel number and assigns the random access memory (RAM) address in each pixel memory group, and the FIFO depth depends upon the number of pixels in each group.

In the scan process, each scan in a group must be performed in accordance with four states. State-0 reads the parameter, which includes focusing and grouping of the parameter for each group, and its duration is related to the number of parameters. State-1 receives and focuses the echo ultrasonic data, and its duration is related to the sampling depth. State-2 is a buffer to wait for the next scan. In state-3, if the focal law is not completed for a group, the process returns to state-0; if the focal law is completed, the pixel data are output to the corresponding FIFO. The state diagram is shown in Fig 3(B). From Fig 3(B), scanning is performed as per the *N*_*FL*_ focal laws in states-0 and 1 between *N*_*FL*_ time conversions. State-2 waits for the next scan and state-3 outputs the focused pixel data. The different focal laws (element numbers), scan depths, and numbers of ROI pixels generate different data start times and volumes of echo data in the related group.

For the *j*-th sensor group, states-0, 1, 2, and 3 correspond to the processing times of $T_{RD}^{j}$, $T_{SD}^{j}$, $T_{wait}^{j}$, and $T_{write}^{j}$, respectively; $T_{RD}^{j}$ is the processing time for each focal law to read the parameter, and is related to the focus parameter and the number of ROI pixels. $T_{SD}^{j}$ is the processing time for ultrasonic echo data acquisition and focus for each focal law; $T_{SD}^{j}=D_{SD}^{j}\times T_{CLK}$, where *D*_*SD*_ is the sample depth and *T*_*CLK*_ is the FPGA clock cycle. $T_{wait}^{j}$ is the wait and system buffering time, which can be set. $T_{write}^{j}$ is the output-pixel-data time (writing to RAM); $T_{write}^{j}=N_{Px}^{j}\times T_{CLK}$, where $N_{Px}^{j}$ is the number of pixels in the ROI. $N_{{}_{FL}}^{j}$ and $N_{e}^{j}$ are the number of focal laws and the number of elements in the *j-*th group. In the TFM system, $N_{{}_{FL}}^{j}=N_{e}^{j}$. The entire scanning sequence diagram in the ModelSim simulation in the TFM ultrasonic system with four elements in one sensor group is depicted in Fig 3(C). A focused image is depicted in Fig 3(D).

**Fig 4:**
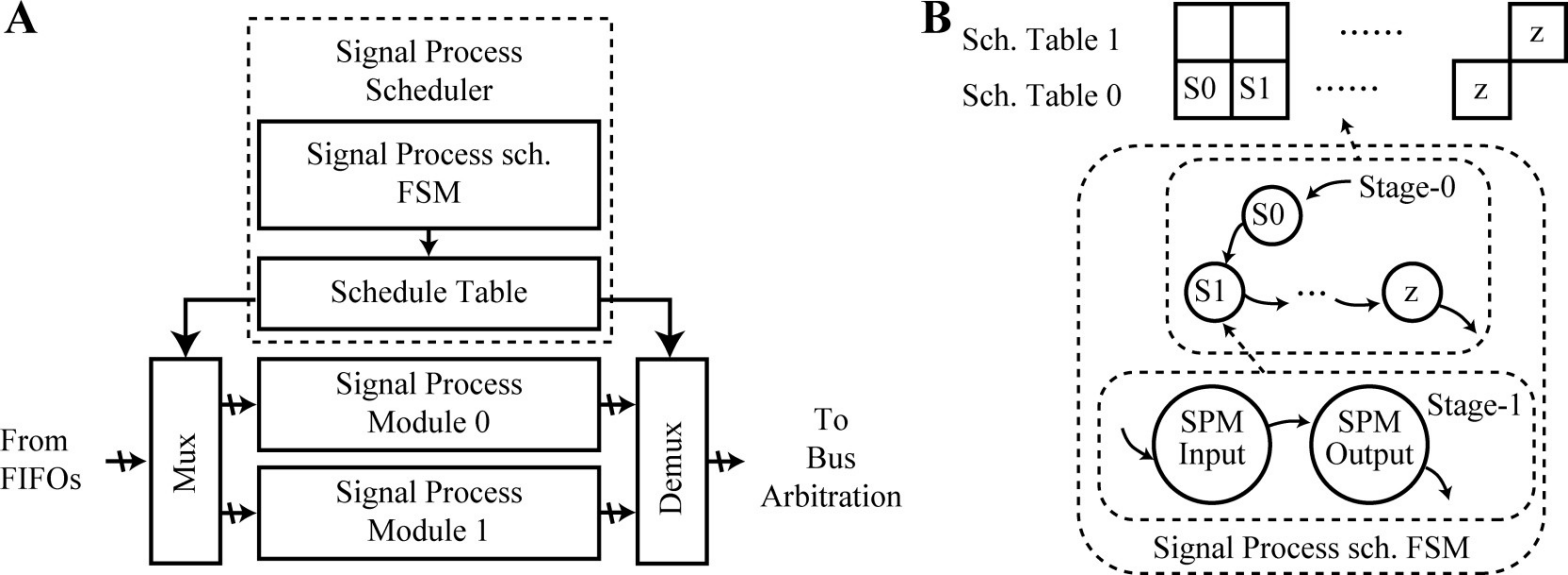
SPA flowchart and architecture. (a) Architecture and (b) signal process schedule FSM flowchart.

Fig 4(A) depicts the signal processing architecture. The SPS includes the signal process schedule finite state machine (FSM) and the scheduler table. It controls the mux and demux in order to determine the time when the pixel signals are output to the FIFOs and input to the SPMs, the mux and demux are dispatched, and the delay control is implemented. According to the schedule table, the states of the signal process schedule FSM control the signal process scheduling in each SPM ensures that the dispatch is implemented in sequence and is not disrupted. The signal process schedule FSM flowchart is depicted in Fig 4(B).

In the SPS process, the mux and demux in the modules require delay control, which can be used to handle the delay in the input and output signals through the SPMs. The mux and demux delays are constant; we label the sum of the mux and demux delays as *T*_*dl*_. The SPM delay is determined according to the structure of the SPMs. For a designed filter and circuit structure, the delay is constant, ensuring the timing.

From the above, the system has a number of parameters; these are transformed into the parameters of the scheduling algorithm in the next section. Our goal is not only to satisfy the scanning with different parameters in multi-group, but also to ensure that they pass through the limited SPM resources by scheduling, without affecting the real-time performance and data transmission.

### Mathematical model

We assume that the asynchronous distributed ultrasonic TFM system described in the previous section, has *M* SPMs and *N* tasks to scan. A system can have multiple sensor groups, and each group can have one or multiple scan tasks; therefore, the number of sensor groups *N*_*g*_ is less than or equal to *N*. In this paper, we only consider *N*_*g*_ = *N*. We replace the “job” of the classical scheduling problem with a “task.” There are *N* tasks ready to be processed at their start times, which must be distributed among the *M* SPMs. A processing schedule must be derived in the scheduler for the tasks assigned to each SPM. Each task has a required processing time, *P*_*j*_, and start time, *S*_*j*_. The optimization criterion is the minimization of the maximum completion time (makespan), among the SPMs.

Similar to the classic scheduling problem, it is generally assumed that at any time, each task can be processed by at most one SPM, and each SPM can process only one task. The other characteristics assumed in this paper are as follows:

1. All the data in all the problems are known deterministically when scheduling is undertaken.
2. There are *N* independent tasks available at the start time.
3. SPMs are available always, if they are not busy, with no breakdowns.
4. A task once started on the SPM must be completed without interruption.

Before the model is presented, we introduce the employed parameters and indices in Table 1.

**Table 1:** Parameters of mixed integer linear programming model.

Symbol	Parameter description
*N*	Number of true tasks to be scheduled
*M*	Number of signal-processing modules
*j*	Task index
*i*	SPM index
*P*_*j*_	Processing time of task *j*
*S*_*j*_	Start time of task *j*

The processing time of the *j*-th task is

$$P_{j}=T_{CLK}\times N_{Px}^{j}.$$ (2)

The start time of the *j-*th task is

$$S_{j}=N_{FL}^{j}\left(T_{RP}^{j}+T_{SD}^{j}+T_{wait}^{j}\right)+T_{write}^{j}+T_{dl}.$$ (3)

The problem can now be formulated as follows:

$$X_{ij}=\left\{ \begin{array}{l} 1\;i\mathrm{th}\;\mathrm{task}\;\mathrm{is}\;\mathrm{scheduled}\;\mathrm{in}j\mathrm{th}\;\mathrm{SPM}\\ 0\;i\mathrm{th}\;\mathrm{task}\;\mathrm{is}\;\mathrm{not}\;\mathrm{scheduled}\;\mathrm{in}j\mathrm{th}\;\mathrm{SPM} \end{array}\right.$$ (4)

$$Z=Min\;C_{\max}.$$ (5)

$$C_{j}=\sum_{i=1}^{N}\sum_{j=1}^{M}\left(P_{j}X_{ij}+KS_{j}\right)$$ (6)

$$s.t.\;\sum_{i=1}^{N}X_{ij}=1.$$ (7)

$$X_{ij}\in \left\{0,1\right\}\;\left(i=1,2,\ldots ,N,j=1,2,\ldots .M\right)$$ (8)

$$S_{i}\geq 0,\;P_{i}\geq 0$$ (9)

$$C_{i}\geq C_{k}+\sum_{j=1}^{M}X_{ij}P_{i}+K\left(S_{i}-S_{k}\right)\;\left(S_{i}\geq S_{k}\right)$$ (10)

$$C_{\max}>C_{j}$$ (11)

K∈[0,1] (12)

Eq (4) defines the variable *X*, while Eq (5) minimizes the maximum completion time (*C*_*max*_), i.e., the makespan, which is the maximum completion time of all SPMs. Constraint (6) provides the definition of *C*_*j*_, while Constraint (7) states that task *i* requires only one SPM. Constraints (8) and (9) define the “0 and 1” and the non-negative variables, respectively. Constraint (10) establishes the relationship between the completion times of tasks *i* and *j* that are assigned to the same machine in a specific SPM. Finally, Constraint (11) defines the makespan and Constraint (12) gives the relationship between the start times of two adjacent tasks in the same SPM.

The total permutation of the problem is as follows (total number of possible solution space combinations):

$$P_{N}^{KM}\left[N-\left(K+1\right)M\right],\;K=\left\lfloor \frac{N}{M}\right\rfloor$$

$$\begin{array}{l} P_{N}^{M}\cdot P_{N-M}^{M}\cdot \cdot \cdot P_{N-\left(K+1\right)M}^{M}\cdot P_{N-KM}^{N-KM}\\ =N\cdot \left(N-1\right)\cdot \left(N-2\right)\cdot \cdot \cdot \left(N-M+1\right)\\ \cdot \left(N-M\right)\cdot \left(N-M-1\right)\cdot \cdot \cdot \left(N-2M+1\right)\\ \;\cdot \cdot \cdot \cdot \cdot \cdot \\ \cdot \left[\left(N-\left(K+1\right)M\right)\right]\cdot \left[N-\left(K+1\right)M-1\right]\cdot \cdot \cdot \left(N-KM+1\right)\\ \cdot \left(N-KM\right)=P_{N}^{KM+1}\\ \;s.t.\;K=\left\lceil \frac{N}{M}\right\rceil \end{array}$$ (13)

The upper bound of this problem is

$$C_{opt}\leq \sum_{i=1}^{N}\left(P_{i}+KS_{i}\right)/F,K\in \left[0,1\right],$$ (14)

and the lower bound is

$$A_{j}=Min\left\{P_{j}+KS_{j}\right\},K\in \left[0,1\right]$$

$$FC_{opt}\geq \sum_{j}A_{j}$$

$$C_{opt}\geq \sum_{j}A_{j}/F.$$ (15)

## Proposed SSPA algorithm

The aim is to effectively solve the problem of the different start times and data quantities in the signal processing scheduling problem for multi group scanning that are generated by the SPM multiplexing and distributed scanning, i.e., JSSP with ST. To achieve this, we propose the SSPA algorithm. This algorithm has four main parameters: the start time, processing time, number of tasks, and number of SPMs. The main concept behind the algorithm is to order the task of the sum of the start and processing times, and to schedule it sequentially.

In task scheduling, a task is scheduled to start in each SPM. If the SPM is idle, the task is deferred backward until it is near the next adjacent task. If the dispatch location is occupied, all other tasks in the SPM are deferred, enabling the dispatch task to be inserted into the SPM for scheduling. Next, the completion times of all the SPMs are compared, and the SPM with the earliest completion time is selected as the dispatch SPM of the task. Finally, after each task is scheduled for the SPMs, the scheduling is completed.

Compared to the conventional FCFS and SPT algorithms, the proposed algorithm has the following advantages:

- The task is deferred backward to the next adjacent task, providing time for scheduling of the next task and increasing SPM utilization.
- Based on the results of the previous scheduling iteration and comparison, the scheduling of the proposed algorithm is confirmed to be optimal.
- The amount of calculation has not increased much.

The algorithm steps are as follows:

1. Add the start time and processing time; the sum is *R*_*j*_.
2. Sort *R*∈{*R*_*j*_,*j* = 1,2,…,*n*} by descending order.
3. Dispatch the first *M* tasks to *M* SPMs, based on the order of *R*.
4. According to the *R* order, dispatch the other *N-M* tasks.
5. Allocate task *T*_*j*_ based on its start time to all the SPMs. If the processing of *T*_*j*_ overlaps the other tasks in the SPMs, defer the other tasks to insert *T*_*j*_. If *T*_*j*_ does not overlap, defer the task as much as possible, near the first task in the SPMs.
6. Compare the maximum completion times of all the allocated SPMs; select the SPM with the minimum completion time and dispatch *T*_*j*_ to this SPM.
7. If there are tasks that are not scheduled, return to 4; otherwise, calculate the maximum completion time of all SPMs, *C*_max_.

**Fig 5:**
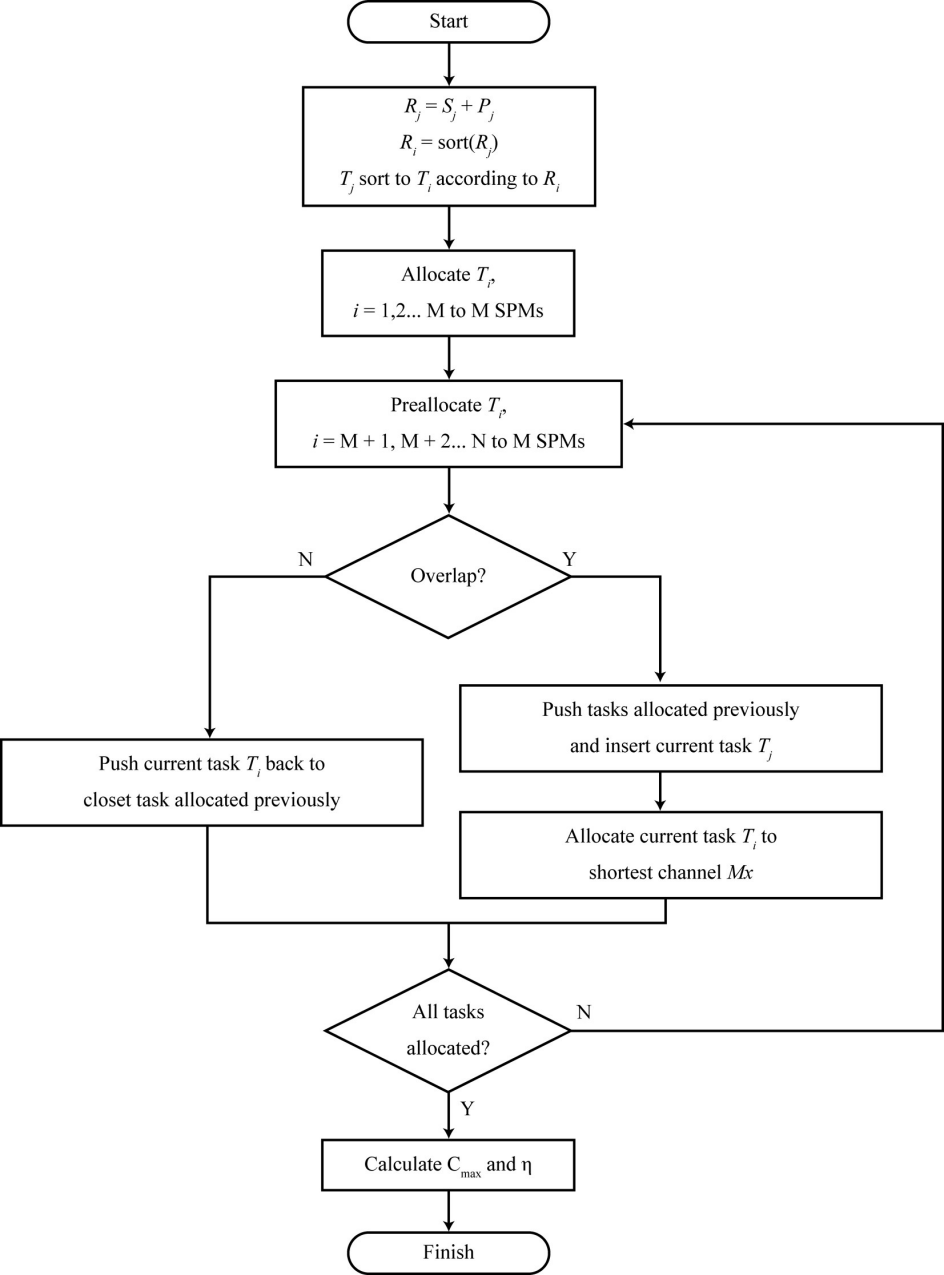
SSPA flowchart.

**Fig 6:**
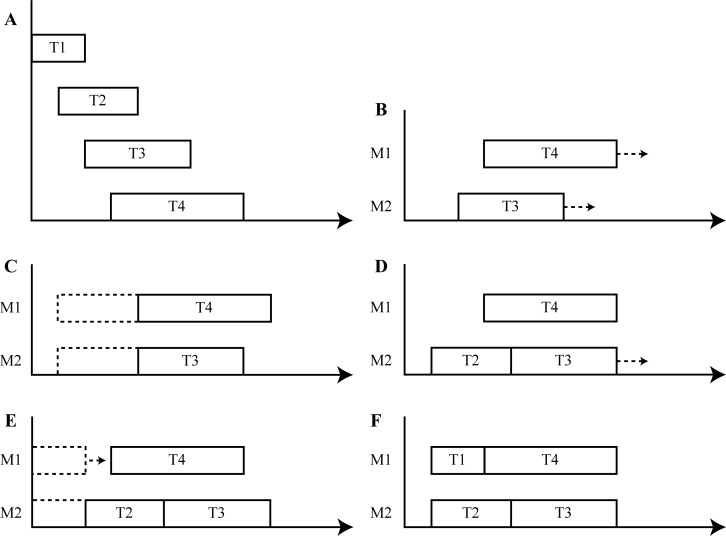
SSPA schematic diagram where M1 and M2 are SPMS and T1−T4 are tasks. (a) Simple example, (b) allocation of T3 and T4, (c) attempted allocation of T2, (d) assignment of T2 to SPM M2, (e) attempted allocation of T1, and (f) completed schedule.

The flowchart and schematic diagram of the proposed SSPA algorithm are shown in Figs 5 and 6, respectively.

We here present a simple example. For tasks T1–T4 depicted in Fig 6(A), the start times are 0, 1, 2, and 3 time units, and the processing times are 2, 3, 4, and 5 time units, respectively. The SPMs are M1 and M2. According to the flow of the SSPA, T3 and T4 are first assigned to M1 and M2, respectively, as depicted in Fig 6(B). Next, allocation of T2 to all SPMs is attempted, as depicted in Fig 6(C). In M1 and M2, T3 and T4 are deferred to insert the dummy T2. M2 is then selected for scheduling of T2, as shown in Fig 6(D). Allocation of dummy task T1 is attempted. If the T1 processing time overlaps the original task in the SPM, the other tasks in the SPM are postponed, e.g., for M2 in Fig 6(E). If there is no overlap, T1 is postponed near the adjacent task, e.g., M1 in Fig 6(E). Finally, the schedule is completed, as depicted in Fig 6(F).

In past literature, the JSSP was taken as NP-hard [41] and associated with one of the most difficult problems in this class [42]. This is because every job in a JSSP can have a different and separate processing time; thus, the problem complexity grows with the number of jobs (tasks). For this reason, JSSP with ST is also an NP-hard problem. The classical SPT algorithm requires sorting and has a time complexity of O(*N*^*2*^). The FCFS algorithm does not need iteration and the time complexity is O(*N*). The SSPA algorithm proposed in this paper first requires sorting; then, the completion times of the SPMs are compared during scheduling. Thus, the time complexity is O(*N*^2^ *M*^2^).

## Experiment results and discussion

We designed a comparison test for the algorithms to prove that the SSPA algorithm enables high utilization and reduces the makespan. The compared algorithms were the FCFS and SPT [43] algorithms, as these are deterministic algorithms, which are often used for processor scheduling. The FCFS algorithm is dispatched to the current free SPM, according to the order of task arrival. The SPT is implemented in accordance with the shortest processing time and is scheduled to the current idle SPM.

In the experiment performed in this study, the start time was taken as being related to the number of focal laws and the scanning depth, and the processing time was taken as being related to the number of pixels in the ROI area; both were independent of each other. The PC used in our experiment was an Intel i7-4970, DDR3 8G RAM, and the programming environment was Matlab 2016a.

In order to evaluate the algorithm performance, we used two criteria: the makespan and the utilization rate. We also varied the four parameters of the algorithms: the start time, processing time, number of tasks, and number of SPMs, so as to study the impact of parameter changes on the algorithm performance.

As noted above, the makespan is defined as the maximum completion time on the SPM after scheduling and is labeled *C*_max_. The makespan data take positive integers and the unit is the FPGA clock-cycle; however, for general purposes, the unit of makespan was taken as the time unit in the following experiments.

The utilization rate is defined as follows:

$$\eta =\frac{\sum_{j}P_{j}}{M\times C_{\max}}\times 100\%,$$ (16)

where *M* is the number of SPMs and *P*_*j*_ is the task processing time. Here, the value was reported as a percentage with two decimal places.

The experiment settings were as follows: The number of SPMs was *M*, and the number of tasks was *N*. Each task had a start time and processing time. The tasks also took a uniform random positive integer within a given range. The FCFS, SPT, and SSPA algorithms were implemented and the values of *M*, *N*, the SPM range, and the task range were changed. All algorithms were executed 100 times, and the average *C*_max_ and utilization rate were calculated. We performed four tests; the parameters are listed in Table 2.

**Table 2:** Experiment settings.

	Upper bound of start time	Upper bound of processing time	Number of tasks	Number of SPMs
**Test 1**	10−100 in steps of 10	100	16	4
**Test 2**	100	10−100 in steps of 10	16	4
**Test 3**	10	10	10–100 in steps of 10	4
**Test 4**	10	10	16	3−10 in steps of 1

In test 1, as detailed in Table 2, the start time was taken as the variable. The start time value range was from 1 to the upper bound in integers, where the upper bound was changed from 10 to 100 in steps of 10. The processing time was set to uniform random integers in the range of 1−100. *N* was 16, and *M* was 4.

**Fig 7:**
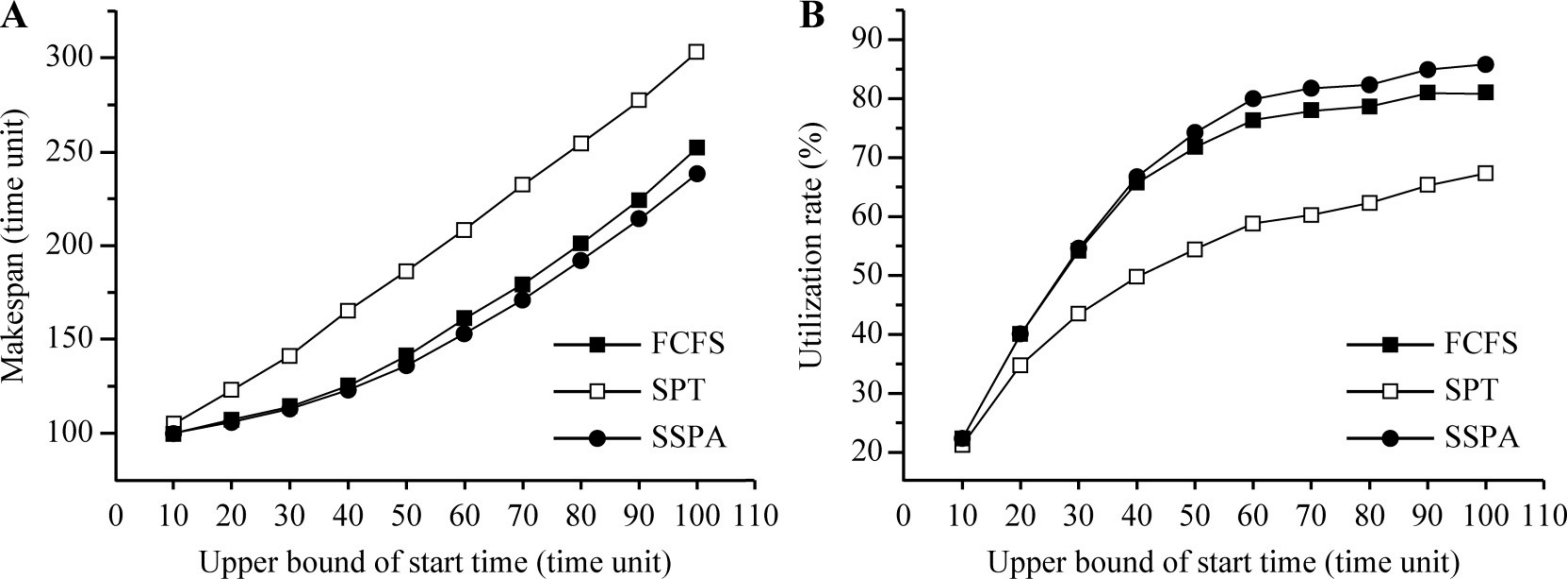
Results for variable upper bound of start time. (a) Makespan and (b) utilization rate.

Increasing the start time will further postpone the task makespan. In test 1, the upper bound of the start time range was varied and the makespan and utilization rates of the three considered algorithms, FCFS, SPT, and SSPA, were compared. The results are listed in Table 3. With the increase in the upper bound, the start time range increased also, and so too did the makespan curve, as shown in Fig 7(A). The SSPA had the smallest makespan, followed by the FCFS. Thus, the SPT makespan was the largest. These results indicate that the SSPA algorithm had the best scheduling performance. The utilization rate increased gradually with the increase in the upper bound and the corresponding gradual increase in the start time range (Fig 7(B)). Gradually, the utilization rate tended to become flat. This is because a bigger ratio between the start time and processing time allows more time slots generated by the start time to be inserted into the small task processing time; hence, a higher utilization rate is achieved. The SSPA utilization rate was always highest in these experiments, followed by those of the FCFS and SPT, as shown in Fig 7(B).

**Table 3:** Results for variable start time.

	Makespan (clock-cycle)			Utilization rate (%)		
Upper bound of start time	FCFS	SPT	SSPA	FCFS	SPT	SSPA
**10**	100	105	100	22.04	21.03	22.04
**20**	107	123	106	39.83	34.56	39.86
**30**	114	141	113	54.07	43.29	54.40
**40**	125	165	123	65.71	49.61	66.62
**50**	141	186	136	71.84	54.26	74.20
**60**	161	208	153	76.32	58.67	79.99
**70**	179	232	171	78.01	60.11	81.78
**80**	201	254	192	78.67	62.18	82.35
**90**	224	277	214	81.03	65.31	84.98
**100**	252	303	238	81.01	67.33	85.86

In test 2, the variable was the processing time, as indicated in Table 2. The processing time range was from 1 to the upper bound in integers, where the upper bound was varied from 10 to 100 in steps of 10. The start time was set to uniform random integers in the range of 1−100. *N* was 16 and *M* was 4.

**Fig 8:**
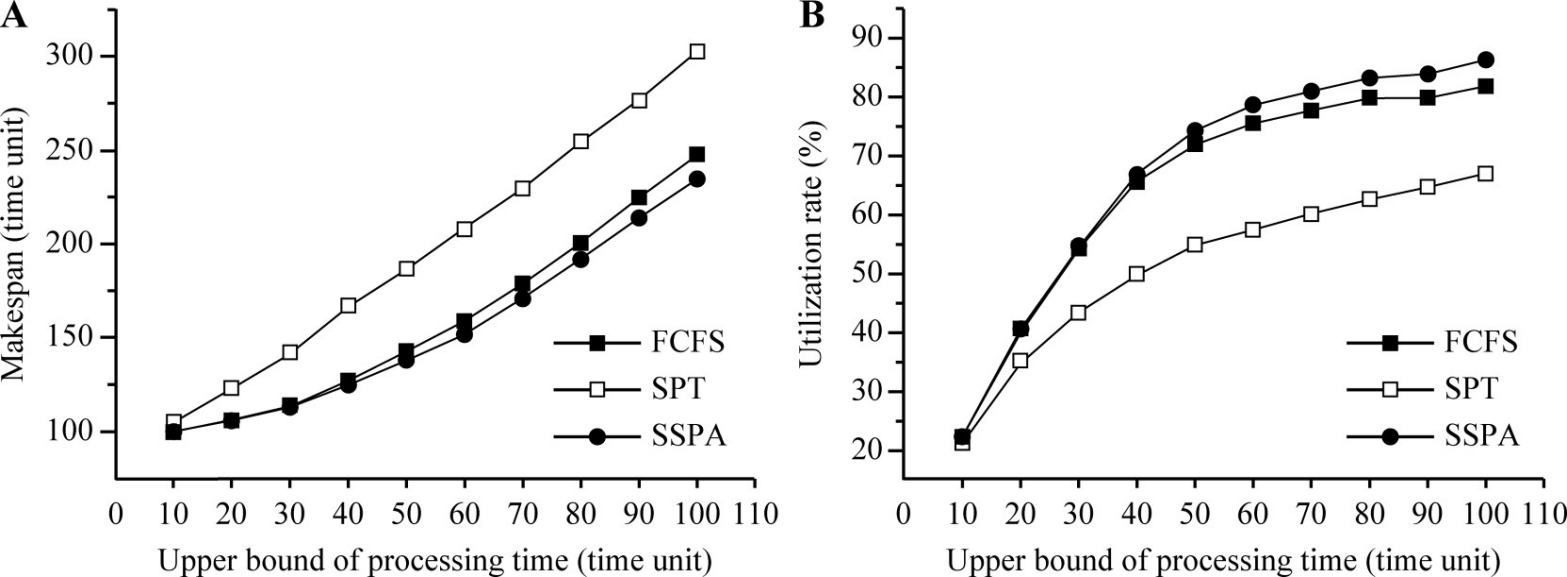
Results for variable upper bound of processing time. (a) Makespan and (b) utilization rate.

Increasing the processing time also delays the task's makespan. In test 2, the upper bound of the processing time range was changed. The makespan and utilization rates of the three algorithms, FCFS, SPT, and SSPA, were compared. The results are listed in Table 4. With the increase in the upper bound, the processing time range increased, along with the makespan curve, as shown in Fig 8(A). The SSPA makespan was always the smallest, followed by the FCFS and SPT. Thus, the SSPA algorithm again exhibited the best scheduling performance. As previously, the utilization rate increased gradually and gradually tended to become flat as the upper bound was increased and the processing time range gradually increased. This is because the processing time of each task was different. Compared to the task with a large processing time, it is easier to insert a task with a small processing time into an idle time slot. Hence, the utilization rate of the problem, which has a small task range, is higher than that of a large tasks range. The SSPA utilization rate was always the highest, followed by that of the FCFS, and finally that of the SPT, as shown in Fig 8(B).

**Table 4:** Results for variable task range.

	Makespan (clock-cycle)			Utilization rate (%)		
Upper bound of processing time	FCFS	SPT	SSPA	FCFS	SPT	SSPA
**10**	100	104	100	22.07	21.10	22.07
**20**	106	123	106	40.58	35.12	40.61
**30**	114	142	113	54.28	43.29	54.70
**40**	127	167	125	65.65	49.95	66.91
**50**	143	187	138	72.13	54.93	74.40
**60**	159	208	152	75.67	57.53	78.81
**70**	179	230	171	77.86	60.19	81.19
**80**	201	255	192	80.01	62.71	83.49
**90**	225	277	214	80.03	64.77	84.10
**100**	248	303	235	82.03	67.10	86.55

In test 3, *N* was changed from 10−100 in steps of 10 (Table 2), the processing time and start time ranges were 1−10 in integers, and *M* was 4.

**Fig 9:**
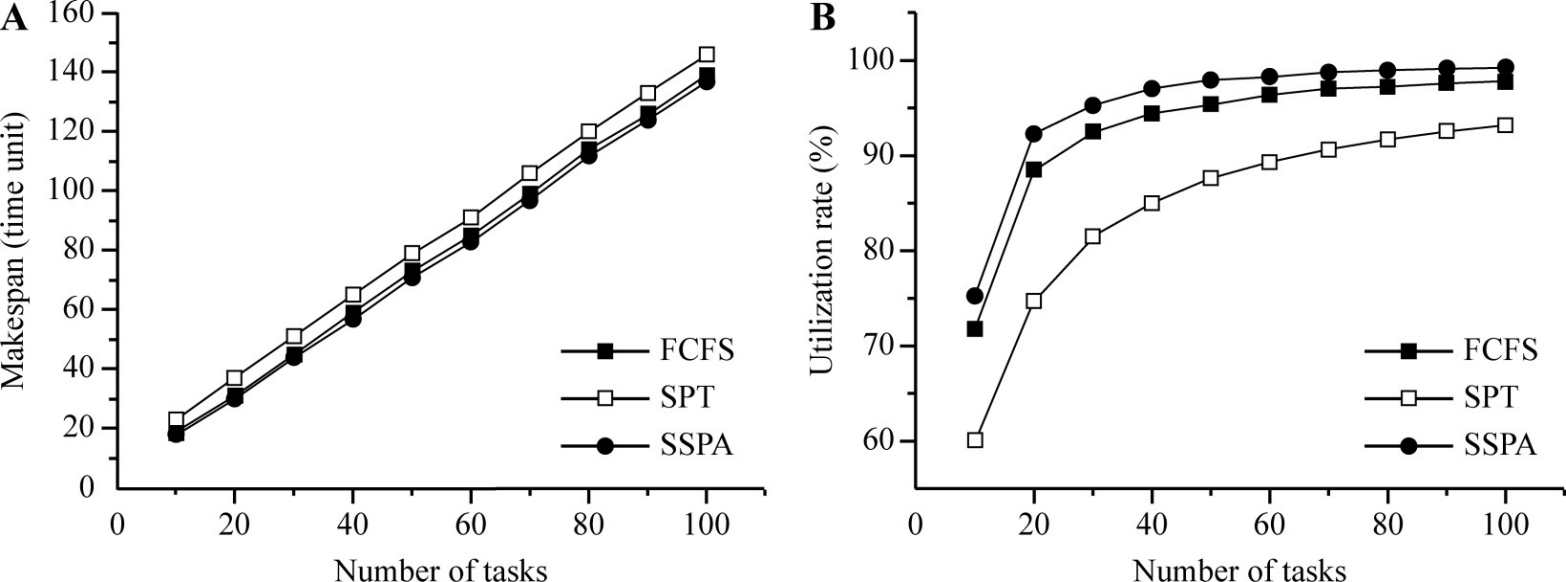
Results for variable number of tasks. (a) Makespan and (b) utilization rate.

Increasing the number of tasks increases the scheduling burden and makespan delay. In Test 3, the number of tasks was increased and the makespan and utilization rates of the three algorithms (FCFS, SPT, and SSPA) were compared. The results are listed in Table 5. The makespan increased linearly with the increase in the number of tasks, as shown in Fig 9(A). The SSPA makespan was always the smallest, followed by those of the FCFS and SPT. The SSPA algorithm exhibited the best scheduling performance, as shown in Fig 9(A). As the number of tasks increased, the utilization rate curve increased and tended to become flat (Fig 9(B)). Furthermore, the utilization rate was higher when the number of tasks increased relative to the number of SPMs. The SSPA utilization rate was always the highest, followed that of the FCFS and finally the SPT, as shown in Fig 9(B).

**Table 5:** Results for variable number of tasks.

	Makespan (clock-cycle)			Utilization rate (%)		
Number of tasks	FCFS	SPT	SSPA	FCFS	SPT	SSPA
**10**	19	23	18	71.74	59.99	75.19
**20**	31	37	30	88.45	74.64	92.27
**30**	45	51	44	92.48	81.47	95.25
**40**	59	65	57	94.43	84.97	97.02
**50**	73	79	71	95.38	87.59	97.91
**60**	85	91	83	96.35	89.31	98.31
**70**	99	106	97	96.96	90.61	98.72
**80**	114	120	112	97.26	91.68	98.95
**90**	126	133	124	97.60	92.53	99.17
**100**	139	146	137	97.88	93.15	99.26

In test 4, *M* was changed from 3−10 in steps of 1 (Table 2), the processing time and start time ranges were 1−10 in integers, and *N* was 16.

**Fig 10:**
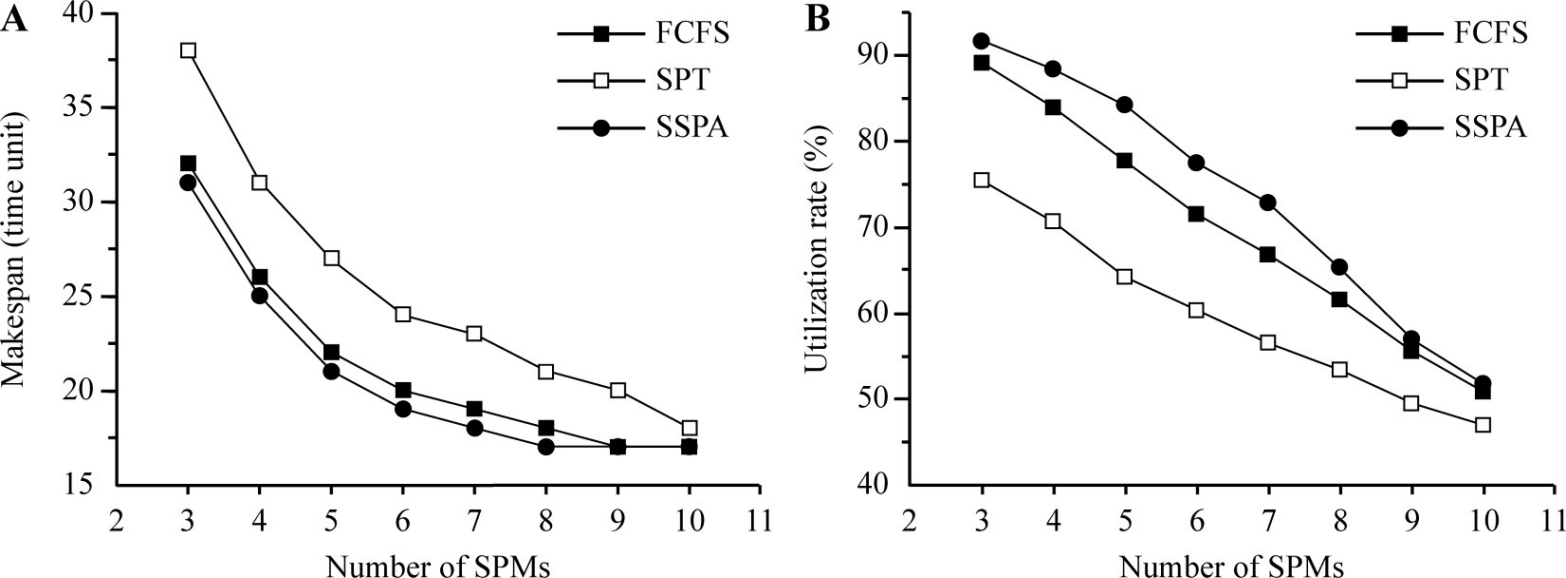
Results for variable number of SPMs. (a) Makespan and (b) utilization rate.

Increasing the SPMs is equivalent to increasing the resources, and the makespan decreases correspondingly. In test 4, the number of SPMs was increased and the makespan and utilization rates of the three algorithms (FCFS, SPT, and SSPA) were compared. The results are listed in Table 6. As the number of SPMs increased, the makespan curve decreased, as shown in Fig 10(A). The SSPA makespan was always the smallest, followed by those of the FCFS and SPT. The SSPA algorithm exhibited the best scheduling performance, as shown in Fig 10(A). As the number of SPMs increased, the curve decreased linearly and the utilization rate decreased continuously. The SSPA utilization rate was always the highest, followed by those of the FCFS and finally the SPT, as shown in Fig 10(B). More resources means that the number of tasks is closer to the number of SPMs; thus, the makespan and utilization rate become more equivalent. That is, if the number of tasks is equal to the number of resources, the makespan of any algorithm takes the maximum completion time of a single task and the utilization rates of the algorithms are equal.

**Table 6:** Results for variable number of SPMs.

	Makespan (clock-cycle)			Utilization rate (%)		
Number of SPMs	FCFS	SPT	SSPA	FCFS	SPT	SSPA
**3**	32	38	31	89.15	75.48	91.72
**4**	26	31	25	83.96	70.69	88.42
**5**	22	27	21	77.74	64.25	84.23
**6**	20	24	19	71.52	60.31	77.51
**7**	19	23	18	66.83	56.58	72.93
**8**	18	21	17	61.59	53.42	65.32
**9**	17	20	17	55.59	49.50	56.99
**10**	17	18	17	50.93	46.93	51.74

Thus, it is established that, compared to the FCFS and SPT algorithms, the SSPA algorithm improves the utilization rate and reduces the makespan.

In addition, a fifth test, test 5, was performed to compare the statistics of the FCFS, SPT, and SPA algorithm experiments. The experimental conditions were as follows: *M* was 4, *N* was 16, the start time was 1−10, the processing time was 1−10, and uniform random integers were selected.

**Fig 11:**
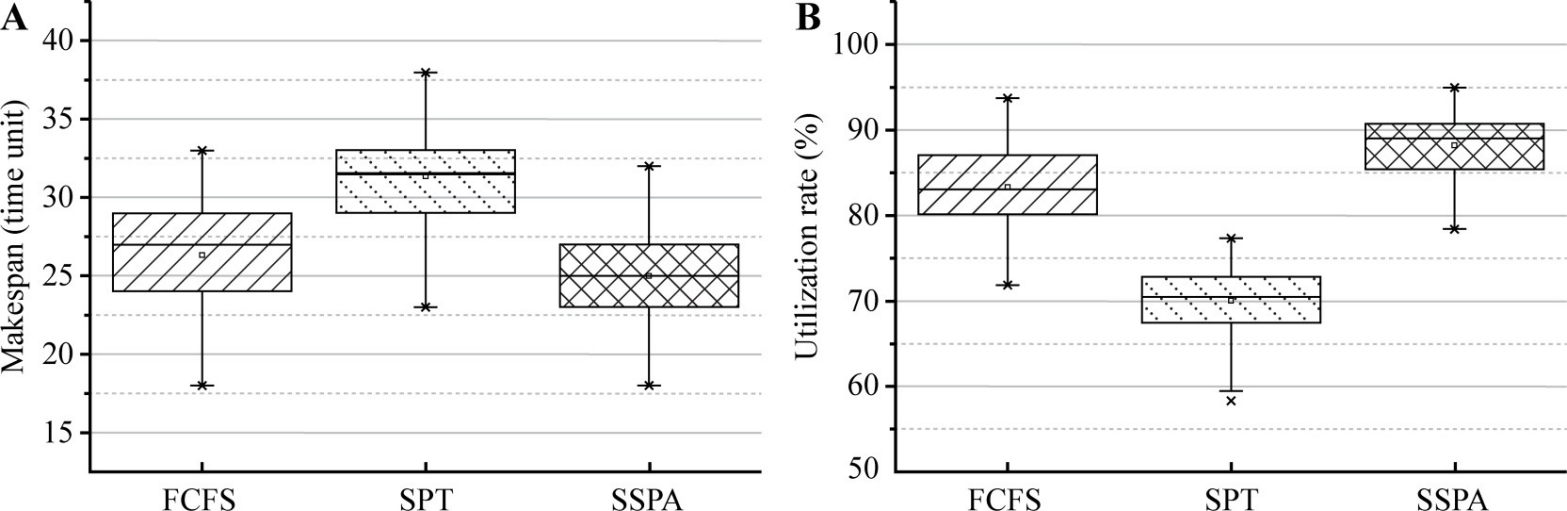
Box plots of FCFS, SPT, and SSPA in JSSP with ST. (a) Makespan and (b) utilization rate.

**Fig 12:**
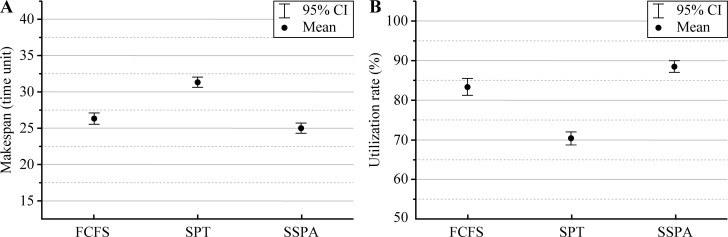
95% CI plots of FCFS, SPT, and SSPA in JSSP with ST. (a) Makespan and (b) utilization rate.

The results for test 5, which compared the statistical performance of the three algorithms (FCFS, SPT and SSPA), are presented in Figs 11 and 12 using box plots and 95% confidence interval plots (95% CI plots), respectively. Similar to the analyses of the makespan and utilization rate, the statistical data of the FCFS, SPT, and SSPA were computed 100 times with given parameters and randomly generated processing and start times. In the box plots, the median and interquartile range (IQR) results were compared. As regards the makespan, the SSPA had the smallest median, the narrowest IQR, and the lowest abnormal upper limit, as shown in Fig 11(A). These results can explain why the SSPA algorithm exhibited the smallest overall makespan. Note that the abnormal data over the upper quartile were also lower in the makespan data set, which indicates the best statistical performance. For the utilization rate comparison, the SSPA had the largest median, which was similar to that of the SPT (Fig 11(B)). The SSPA algorithm had the smallest IQR, the largest abnormal lower limit, and the smallest abnormal range, as shown in Fig 11(B); these results indicate that the SSPA algorithm yields more centralized data, and that the overall data has the highest utilization rate and fewer abnormal points compared to those of the other two algorithms.

From the 95% CI plots shown in Fig 12, the SSPA had the minimum mean value for both the makespan and utilization rate. Furthermore, the three algorithms had almost identical confidence intervals. Compared with FCFS, the SPT algorithm had the best performance in the 95% CI plot, as shown in Fig 12.

Finally, Table 7 compares the average elapsed times of the three algorithms for the test 5 calculation. Although the average elapsed time of the SSPA calculation was greater than those of the FCFS and SPT algorithms, it is acceptable in that it is of the same order of magnitude.

**Table 7:** Elapsed times for FCFS, SPT, and SSPA in JSSP with ST.

	FCFS	SPT	SSPA
**Avg. Time (ms)**	45.53	15.76	66.51

### Scheduling algorithm implementation and performance evaluation

The scheduling algorithm was implemented using a UPA instrument (PA2000 model, Guangzhou Doppler Electronic Technologies Co., Ltd., Guangzhou, China), and a Cyclone V GT FPGA development board (Intel Corporation, Santa Clara, CA., USA) [44,45]as the PCI-E communication module with the PC. The UPA data were transmitted to the PC through the PCI-E interface, and the multi group scanning images were processed. The SPM delay was 15 clock cycles and the main clock frequency was 100 MHz (10 ns per clock cycle).

To better demonstrate the performance of the scheduling algorithm, a virtual scheduling process was used to test the application of the SSPA algorithm in the SPM in this experiment, as per the parameter settings listed in Table 8, corresponding to the symbol in the sub-section asynchronous distributed ultrasonic TFM system architecture. The reading parameter time was approximately converted from the DDR3 hard core frequency of 400 MHz, with 16-bit width and 0.8 efficiency. The sample depth and number of ROI pixels could be directly converted to $T_{SD}^{j}$ and $T_{write}^{j}$. To make the effect more obvious, the sums of the read parameter time ($T_{RD}^{j}$) values and the wait time ($T_{Wait}^{j}$) values in the same group were made to be equal. Finally, we calculated the start and processing times based on Eqs (2) and (3), respectively.

**Table 8:** Parameters used in FPGA implementation.

	Number of elements ($N_{e}^{j}$)	Read parameter time ($T_{RD}^{j}$)	Sample depth ($D_{SD}^{j}$)	Number of ROI pixels ($N_{Px}^{j}$)	Wait tme ($T_{Wait}^{j}$)	Delay time (*T*_*dl*_)
**Group 1**	4	4	1024	64	12	2
**Group 2**	4	5	1024	96	11	2
**Group 3**	4	7	1024	128	9	2
**Group 4**	4	8	1024	160	8	2

**Fig 13:**
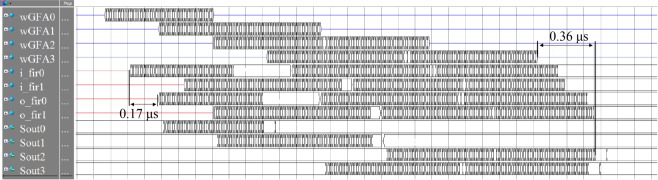
Pre-synthesis simulation results.

Fig 13 displays the results of the pre-synthesis simulation in SPA, using ModelSim 10.2 SE EDA tools (Mentor Co., Ltd., Wilsonville, OR, USA); the inputs to SPM0 and SPM1 were i_fir1, i_fir2, and the outputs to SPM1 and SPM2 were o_fir1, o_fir2, respectively. It can also be seen from the input signals through SPM0 and SPM1 and the output for the SPM delay that the input signals in0, in1, in2, and in3 in the SPA and signal processing were correctly restored as the output signals out0, out1, and out2, respectively. It is also apparent that the delay between the SPM input and output was 17 clock cycles, and the SPM delay was 15 cycles; hence, only a two-clock-cycle delay occurred owing to the mux and demux delays. In addition, it can be observed that the delay between the signal input to the FIFO after focus and the SPA output was only 36 clock cycles; subtracting the 15 clock cycles of the SPM delay, the delay owing to the entire scheduler architecture was approximately 19 clock cycles. Compared to the other time consumption, the delay caused by the SPA architecture was negligible; however, it improved the utilization rate of the SPM considerably.

**Fig 14:**
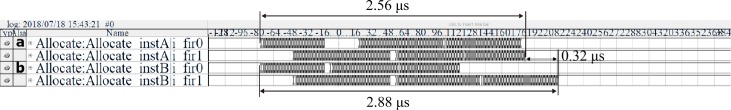
Results for four-pixel signal scheduling in two SPMs using Signaltap II. (a) SSPA algorithm and (b) FCFS or SPT algorithm.

Fig 14 shows a comparison of the FCFS and SSPA algorithms. Four signals were scheduled in two SPMs using Signaltap II, Quartus 13.0 (Intel Corporation, Santa Clara, CA., USA). The SPT/FCFS makespan was 2.88 μs, while that for the SSPA was 2.56 μs; thus, the makespan of the SSPA algorithm was lower. It is apparent that a time saving of 0.32 μs, or 32 clock cycles, was achieved for the same signal using SSPA. Therefore, a makespan improvement of approximately 11% was achieved and the utilization rate was improved by approximately 9.72%.

## Conclusions and future research

A novel algorithm called the SSPA was proposed, which is based on a scheduler realized using FPGA technology. The SSPA algorithm was applied to an asynchronous distributed ultrasonic TFM system; hence, the bandwidth utilization was maximized by 9.72% while the makespan was reduced by 11% compared to the conventional FCFS and SPT algorithms. The system could also flexibly group the array without restricting the number of elements in each group.

A mathematical model of the problem was established, and the total permutation of the problem and its upper and lower bounds were indicated. The steps and procedures of the SSPA algorithm were presented and its performance was demonstrated.

To improve the transmission efficiency of the considerable volume of data generated by an asynchronous distributed ultrasonic TFM system and the real-time performance of algorithms realized using FPGA technology, the SSPA scheduling algorithm, based on SPM, has significant potential for application in multi-sensor systems. Future research is likely to focus on the design of certain special scheduling algorithm modules for different sensor systems, or on study of the scheduling problem of distributed ultrasonic systems based on multi-FPGA technology.

## Supporting information

S1 Appendix Multigroup data. (XLSX)

## References

[1] McNab A and Campbell MJ. Ultrasonic phased arrays for nondestructive testing. NDT Int. 1987:20 333–337. 10.1016/0308-9126(87)90290-2

[2] Jensen JA, Nikolov SI, Gammelmark KL, Pedersen MH. Synthetic aperture ultrasound imaging. Ultrasonics 2006;44: e5–e15. 10.1016/j.ultras.2006.07.017 16959281

[3] Holmes C, Drinkwater BW, Wilcox PD. Post-processing of the full matrix of ultrasonic transmit-receive array data for non-destructive evaluation. NDT & E Int. 2005;38: 701–711. 10.1016/j.ndteint.2005.04.002

[4] Njiki M, Bouaziz S, Elouardi A, Casula O, Roy O. A multi-FPGA implementation of real time reconstruction using total focusing method. 2013 IEEE 3rd Annual International Conference on Cyber Technology in Automation, Control and Intelligent Systems (CYBER), Nanjing, China. 2013; 468–473. 10.1109/cyber.2013.6705491

[5] Taylor KJ, Milan J. Differential diagnosis of chronic splenomegaly by grey-scale ultrasonography: Clinical observations and digital A-scan analysis. Br J Radiol. 1976;49: 519–525. 10.1259/0007-1285-49-582-519 1276625

[6] Wang C, Mao J, Leng T, Zhuang ZY, Wang XM. Efficient acceleration for total focusing method based on advanced parallel computing in FPGA. Int J Acoust Vib. 2017;22: 536–540. 10.20855/ijav.2017.22.4500

[7] Li Y, Blalock TN, Hossack JA. Synthetic axial acquisition-full resolution, low-cost C-scan ultrasonic imaging. IEEE Trans Ultrason Ferroelectr Freq Control. 2008;55: 236–239. 10.1109/TUFFC.2008.632 18334329

[8] Lin RB, Liu GX, Tang WM. FPGA implementation of ultrasonic s-scan coordinate conversion based on radix-4 CORDIC algorithm. Int J Eng Technol. 2015;7: 249–253. 10.7763/ijet.2015.v7.800

[9] Bank Dirk, and Kampke T. "High-Resolution Ultrasonic Environment Imaging." IEEE Trans Robotics 23 2(2007):370–381. 10.1109/TRO.2007.895060

[10] Jifei H J H, Xiang L X L, Dashun Y D Y. A multisensor fusing system on ultrasonic sensors[C]// Vehicle Electronics Conference. IEEE, 1999.doi: 10.1109/IVEC.1999.830645

[11] Ang W, Juanhua Z, Fuyun H, Jiandong H, Ling W. Measuring system for the wall thickness of pipe based on ultrasonic multisensor[C]// International Conference on Electronic Measurement & Instruments. IEEE, 2009.doi: 10.1109/ICEMI.2009.5274785

[12] Song J, Pulkkinen A, Huang Y, Hynynen K. 2012 Hynynen, Investigation of standing-wave formation in a human skull for a clinical prototype of a large-aperture, transcranial MR-guided focused ultrasound (MRgFUS) phased array: An experimental and simulation study. IEEE Trans Biomed Eng. 2012;59: 435–444. 10.1109/TBME.2011.2174057 22049360 PMC4095975

[13] ASTM F2491-13. 2013 Standard guide for evaluating performance characteristics of phased-array ultrasonic testing instruments and systems ASTM: West Conshohocken, PA, USA.

[14] Adams J, Balas E, Zawack D. The shifting bottleneck procedure for job shop scheduling. Manag Sci. 1987;34: 391–401 10.1287/mnsc.34.3.391

[15] Balas E. Machine sequencing via disjunctive graphs: an implicit enumeration algorithm. Oper Res. 1969;17: 927–1092. 10.1287/opre.17.6.941

[16] Jackson JR. Scheduling a production line to minimize maximum tardiness. Research Report 43, Management Science Research Projects. Los Angeles University of California Management Sciences Research Project: USA; 1955.

[17] Smith W. E. (2010). Various optimizers for single-stage production. Naval Research Logistics Quarterly, 3(1–2), 59–66. 10.1002/nav.3800030106

[18] Wang SF, Zou YR. Techniques for the Job Shop Scheduling Problem: a Survey. System Engineering Theory and Practice. 23(1):49–55. 10.3321/j.issn:1000-6788.2003.01.009

[19] Panwalkar SS, Iskander W. A survey of scheduling rules. Oper Res. 1977;25: 45–61. 10.1287/opre.25.1.45

[20] Wu, S.Y.D. An expert systems approach for the control and scheduling of flexible manufacturing systems. Ph.D. Thesis. Pennsylvania State University. 1987. Available from: https://www.osti.gov/biblio/6963868

[21] Ying KC, Lin SW. Minimizing makespan for no-wait flowshop scheduling problems with setup times. Comput Ind Eng. 2018;121: 73−81. 10.1016/j.cie.2018.05.030

[22] Lin SW, Huang CY, Ying KC, Chen DL. Decreasing the system testing makespan in a computer manufacturing company. IEEE Access 2018;6: 16464−16473. 10.1109/ACCESS.2018.2816959

[23] Suzuki A, Morie T, Tamukoh H. A shared synapse architecture for efficient FPGA implementation of autoencoders. PLOS ONE 2018;13 3: e0194049 10.1371/journal.pone.0194049 29543909 PMC5854352

[24] Rodríguez-Flores L, Morales-Sandoval M, Cumplido R, Feregrino-Uribe C, Algredo-Badilo I. Compact FPGA hardware architecture for public key encryption in embedded devices. PLOS ONE 2018;13 1: e0190939 10.1371/journal.pone.0190939 29360824 PMC5779673

[25] Hossain MS, Saeedi E, Kong Y. Parallel point-multiplication architecture using combined group operations for high-speed cryptographic applications. PLOS ONE 2017;12 5: e0176214 10.1371/journal.pone.0176214 28459831 PMC5411040

[26] Kim HJ, Choi KI. A pipelined non-deterministic finite automaton-based string matching scheme using merged state transitions in an FPGA. PLOS ONE 2016;11 10: e0163535 10.1371/journal.pone.0163535 27695114 PMC5047626

[27] Srinivasan S, Pandharipande A. 2013 Self-configuring scheduling protocol for ultrasonic sensor systems. IEEE Sens J. 2013;13: 2517–2518. 10.1109/jsen.2013.2254594.

[28] Patil S, Kulkarni RA, Patil SH, Balaji N. Performance improvement in cloud computing through dynamic task scheduling algorithm. In Proceedings of the International Conference on Next Generation Computing Technologies, Dehradun, India, 2005: 96–100. 10.1109/ngct.2015.7375090

[29] Chronaki K, Rico A, Casas M, Moreto M, Badia R, Ayguade E. 2017 Task scheduling techniques for asymmetric multi-core systems IEEE Trans Parallel Distrib Syst. 2015;28: 2074–2087. 10.1109/TPDS.2016.2633347

[30] Long J, Dong M, Ota K, Liu A. A green TDMA scheduling algorithm for prolonging lifetime in wireless sensor networks. IEEE Syst. J. 2017;11: 868–877. 10.1109/JSYST.2015.2448355

[31] Yaashuwanth C, Ramesh RA. A new scheduling algorithm for real time system. Int J Comp Sci Inf Secu. 2009;6: 61–66.

[32] Liu J, Soleimanifar M, Lu M. Resource-loaded piping spool fabrication scheduling: Material-supply driven optimization Visual. Eng. 2017;5: 1–14. 10.1186/s40327-017-0044-3

[33] Fischetti M, Monaci M. Using a general-purpose mixed-integer linear programming solver for the practical solution of real-time train rescheduling. Eur J Oper Res. 2017;263: 258–264. 10.1016/j.ejor.2017.04.057

[34] Ou X, Chang Q, Chakraborty N, Wang J. Gantry scheduling for multi-gantry production system by online task allocation method. IEEE Robot Autom Lett. 2017;99: 1848–1855. 10.1109/lra.2017.2710259

[35] Tang W, Liu G, Li Y, Tan D. An improved scheduling algorithm for data transmission in ultrasonic phased arrays with multi-group ultrasonic sensors Sensors. 2017;17: 2355 10.3390/s17102355 29035345 PMC5676639

[36] Caicedo D, Pandharipande A. Ultrasonic arrays for localized presence sensing. IEEE Sens J. 2012;12: 849–858. 10.1109/JSEN.2011.2161667

[37] Priyantha NB, Chakraborty A, Balakrishnan H. The cricket location-support system. Proc ACM Int Conf Mobile Computing and Networking. 2000; 32–43. 10.1145/345910.345917

[38] Caicedo D, Pandharipande A. Distributed ultrasonic zoned presence sensing system. IEEE Sens J. 2014;14: 234–243. 10.1109/JSEN.2013.2282958

[39] Zhang F, Chen J, Li H, Sun Y, Shen X. Distributed active sensor scheduling for target tracking in ultrasonic sensor networks. ACM Mobile Netw Appl. 2011;34; 1771–1772. 10.1007/s11036-011-0311-9

[40] Caicedo D, Pandharipande A. Ultrasonic array sensor for indoor presence detection. In Proceedings of the 20th European Signal Processing Conference (EUSIPCO) 2012, Bucharest, Romania.

[41] Garey MR, Johnson DS. Computers and Intractability: A Guide to the Theory of NP-Completeness, W. H. Freeman, San Francisco, Calif, USA, 1979.

[42] Takeshi Y, Ryohei N. Job shop scheduling. IEE Control Engineering Series. Heidelbergy, Berlin, Germany, 1997;55: 134–160. 1997

[43] Pinedo ML. Scheduling: Theory, Algorithms, and Systems. Springer Berlin Heidelberg, 2016 10.1080/15458830.1996.11770714

[44] Cyclone V Avalon-ST Interface for PCIe Solutions User Guide. [cited 15 Jan 2017] Available online: https://www.intel.com/content/dam/altera-www/global/en_US/pdfs/literature/ug/ug_c5_pcie_avst.pdf

[45] SCFIFO and DCFIFO IP Cores User Guide. Available online: https://www.altera.com/content/dam/alterawww/global/en_US/pdfs/literature/ug/ug_fifo.pdf (accessed on 2 January 2017)

